# Absolute treatment effects of novel antidiabetic drugs on a composite renal outcome: meta-analysis of digitalized individual patient data

**DOI:** 10.1007/s40620-023-01858-8

**Published:** 2024-01-18

**Authors:** Maximilian Brockmeyer, Claudio Parco, Kris Gregory Vargas, Ralf Westenfeld, Christian Jung, Malte Kelm, Michael Roden, Cihan Akbulut, Sabrina Schlesinger, Georg Wolff, Oliver Kuss

**Affiliations:** 1 Division of Cardiology, Pulmonology and Vascular Medicine, Medical Faculty and University Hospital Düsseldorf, Heinrich-Heine-University Düsseldorf, Düsseldorf, Germany; 2 Nuffield Department of Population Health, University of Oxford, Oxford, UK; 3 Cardiovascular Research Institute Düsseldorf (CARID), Heinrich-Heine-University Düsseldorf, Düsseldorf, Germany; 4 Division of Endocrinology and Diabetology, Medical Faculty and University Hospital Düsseldorf, Heinrich-Heine-University Düsseldorf, Düsseldorf, Germany; 5 Institute for Clinical Diabetology, German Diabetes Center, Leibniz Center for Diabetes Research at Heinrich-Heine-University Düsseldorf, Düsseldorf, Germany; 6 German Center for Diabetes Research, Partner Düsseldorf, Munich-Neuherberg, Germany; 7 Institute for Biometrics and Epidemiology, German Diabetes Center, Leibniz Center for Diabetes Research at Heinrich-Heine-University Düsseldorf, Düsseldorf, Germany; 8 Center for Health and Society, Faculty of Medicine, Heinrich Heine University Düsseldorf, Düsseldorf, Germany; 9 Division of Cardiology, Pulmonology and Vascular Medicine, Department of Conservative Medicine, Heinrich-Heine-University Düsseldorf, Moorenstr. 5, 40225 Düsseldorf, Germany

**Keywords:** Absolute treatment effect, Composite renal outcome, GLP-1 receptor agonist, SGLT2 inhibitor

## Abstract

**Background:**

Absolute treatment benefits—expressed as numbers needed to treat—of the glucose lowering and cardiovascular drugs, glucagon-like peptide-1 (GLP-1) receptor agonists and sodium-glucose transporter 2 (SGLT2) inhibitors on renal outcomes remain uncertain. With the present meta-analysis of digitalized individual patient data, we aimed to display and compare numbers needed to treat of both drugs on a composite renal outcome.

**Methods:**

From Kaplan–Meier plots of major cardiovascular outcome trials of GLP-1 receptor agonists and SGLT2 inhibitors vs. placebo, we digitalized individual patient time-to-event information on composite renal outcomes with WebPlotDigitizer 4.2; numbers needed to treat from individual cardiovascular outcome trials were estimated using parametric Weibull regression models and compared to original data. Random-effects meta-analysis generated meta-numbers needed to treat with 95% confidence intervals (CI).

**Results:**

Twelve cardiovascular outcome trials (three for GLP-1 receptor agonists, nine for SGLT2 inhibitors) comprising 90,865 participants were included. Eight trials were conducted in primary type 2 diabetes populations, two in a primary heart failure and two in a primary chronic kidney disease population. Mean estimated glomerular filtration rate at baseline ranged between 37.3 and 85.3 ml/min/1.73 m^2^. Meta-analyses estimated meta-numbers needed to treat of 85 (95% CI 60; 145) for GLP-1 receptor agonists and 104 (95% CI 81; 147) for SGLT2 inhibitors for the composite renal outcome at the overall median follow-up time of 36 months.

**Conclusion:**

The present meta-analysis of digitalized individual patient data revealed moderate and similar absolute treatment benefits of GLP-1 receptor agonists and SGLT2 inhibitors compared to placebo for a composite renal outcome.

**Graphical Abstract:**

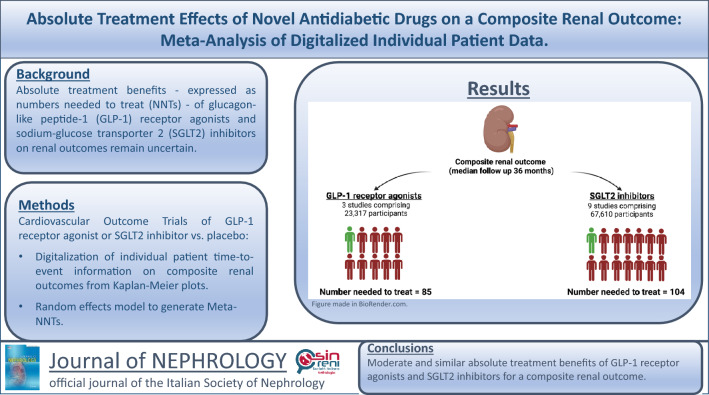

**Supplementary Information:**

The online version contains supplementary material available at 10.1007/s40620-023-01858-8.

## Introduction

Type 2 diabetes mellitus (T2DM), with prevalence increasing in many world regions, is a major global health issue [1, 2]. As a significant cause of cardiovascular disease and chronic kidney disease (CKD) [3, 4], T2DM is associated with a great burden of morbidity and mortality and subsequent high socio-economic impact [5, 6]. Thus, optimal treatment of T2DM and associated comorbidities is crucial to reduce the global burden of disease.

Updated T2DM treatment guidelines promote the use of sodium-glucose transporter 2 (SGLT2) inhibitors as well as glucagon-like peptide-1 (GLP-1) receptor agonists in subjects at high cardiovascular risk or with known cardiovascular disease [7, 8]. This recommendation derives from beneficial effects on diabetes-related cardiovascular as well as renal outcomes in major cardiovascular outcome trials in subjects with T2DM [9, 10]. Subsequently, these glucose-lowering drugs were shown to be effective also in populations without T2DM. As a result, SLGT2 inhibitors (empagliflozin and dapagliflozin) are now an integral part of first-line therapy in heart failure with reduced ejection fraction [11–14], and are the first drug class ever showing statistically significant reductions in major cardiovascular events in heart failure with preserved ejection fraction [15, 16]. GLP-1 receptor agonists (liraglutide and semaglutide) adopt an emerging role in specific treatment of obesity, showing sustained effects on weight loss in large scale trials [17].

For approval of antidiabetic therapy to treat T2DM, the U.S. Food and Drug Administration requires proof of non-inferiority on the hazard ratio (HR). Therefore, the majority of reports from cardiovascular outcome trials give only these relative effect estimates. Respective meta-analyses pooling data from these trials mostly report relative effects as well [10, 18]. In contrast, although it is recommended in guidelines, absolute treatment effects have rarely been reported in trials. Expression of absolute treatment effects by numbers needed to treat provides fundamental advantages in evaluating the cost/benefit ratio of a drug intervention. They help to clarify medical beneficial potential and allow evaluation of economic implications [19–21]. Since patient-level data from individual trials are not publicly available in the majority of cases, meta-analyses of absolute treatment effects for time-to-event outcomes cannot be performed. In order to incorporate absolute treatment effects on renal outcomes in a comprehensive meta-analysis, we applied a validated method of digitalization of time-to-event information obtained from publications of cardiovascular outcome trials investigating GLP-1 receptor agonists and SGLT2 inhibitors [22–26], as previously applied to cardiovascular and mortality outcomes [27, 28].

## Methods

### Study selection, outcome definition, data extraction and quality assessment

Eligible trials comparing SGLT2 inhibitors or GLP-1 receptor agonists to placebo were identified from the cardiovascular outcome trial summit reports by Schnell et al. [29–36], annual reflections on all major randomised controlled trials investigating cardiovascular outcomes in the field of diabetes and associated diseases. The most recent cardiovascular outcome trial summit report was published in March 2023. To identify relevant cardiovascular outcome trials outside of the regular cardiovascular outcome trial summit reports, we cross-checked a well known expert forum report on cardiovascular outcome trials in T2DM by Cefalu et al. [37]. However, no additional cardiovascular outcome trials were found. No protocol was pre-registered (e.g. in PROSPERO).

Full texts of cardiovascular outcome trial reports as well as supplementary information were searched for Kaplan–Meier plots depicting time-to-event information for a composite renal outcome*.* Cardiovascular outcome trials were excluded when no Kaplan–Meier plot for a composite renal outcome of SGLT2 inhibitors or GLP-1 receptor agonists vs. placebo were retrievable. Individual trial definitions of composite renal outcomes as reported served as the primary endpoint; details are provided in Table 1.

**Table 1:** Individual trials with their definitions of composite renal outcomes

Study	Journal and year	Study drug	Composite renal outcome definition
GLP-1 receptor agonists			
AMPLITUDE-O [41]	NEJM 2021	Efpeglenatide	Incident macroalbuminuria defined as a urine albumin-creatinine ratio > 33.9 mg/mmol with ≥ 30% rise from baseline, decline in eGFR from baseline of ≥ 40% for ≥ 30 days, renal replacement therapy for ≥ 90 days, or incident eGFR < 15 ml/min/1.73 m^2^ for ≥ 30 days
LEADER [42, 43]	NEJM 2016	Liraglutide	Incident macroalbuminuria, sustained doubling of the serum creatinine level, eGFR of ≤ 45 mL/min/1.73m^2^, renal-replacement therapy with no reversible cause, or renal death
REWIND [44, 45]	Lancet 2019	Dulaglutide	Incident macroalbuminuria defined as a urine albumin-creatinine ratio > 33.9 mg/mmol, sustained decline in eGFR from baseline of ≥ 30%, or renal replacement therapy
SGLT2 inhibitors			
CANVAS [46]	NEJM 2017	Canagliflozin	Sustained doubling of serum creatinine from baseline, incident end-stage kidney disease, or renal death
CREDENCE [47]	NEJM 2019	Canagliflozin	Incident end-stage kidney disease, doubling of the serum creatinine level from baseline for ≥ 30 days, or renal or cardiovascular death
DAPA-CKD [48]	NEJM 2020	Dapagliflozin	Decline in the eGFR from baseline of ≥ 50% for ≥ 28 days, incident end-stage kidney disease, or renal death
DECLARE-TIMI58 [49]	NEJM 2019	Dapagliflozin	Sustained decline in the eGFR from baseline of ≥ 40% to < 60 ml/min/1.73 m^2^, incident end-stage renal disease, or renal or cardiovascular death
EMPA-KIDNEY [50]	NEJM 2023	Empagliflozin	Sustained decline in the eGFR from baseline of ≥ 40%, sustained eGFR < 10 mL/min/1.73m^2^, incident end-stage kidney disease, or renal death
EMPA-REG [51, 52]	NEJM 2015	Empagliflozin	Doubling of the serum creatinine, renal replacement therapy, or renal death
EMPEROR-PRESERVED [15, 53]	NEJM 2021	Empagliflozin	Sustained decline in the eGFR from baseline of ≥ 40%, chronic renal replacement therapy, renal transplant, or a sustained eGFR < 15 mL/min/1.73 m^2^ (for persons with baseline eGFR ≥ 30) or sustained eGFR < 10 mL/min/1.73m^2^ (for persons with baseline eGFR < 30 mL/min/1.73m^2^)
EMPEROR-REDUCED [12, 53]	NEJM 2020	Empagliflozin	Sustained decline in the eGFR from baseline of ≥ 40%, chronic renal replacement therapy, renal transplant, or a sustained eGFR < 15 mL/min/1.73 m^2^ (for persons with baseline eGFR ≥ 30) or sustained eGFR < 10 mL/min/1.73m^2^ (for persons with baseline eGFR < 30 mL/min/1.73m^2^)
VERTIS-CV [54]	NEJM 2020	Ertugliflozin	Sustained doubling of the serum creatinine, renal replacement therapy, or renal death

Individual trials with their definitions of composite renal outcomes

*GLP-1* glucagon-like peptide 1, *eGFR* estimated glomerular filtration rate, *NEJM* New England Journal of Medicine, *SGLT2* sodium glucose transporter 2

Digitalization of individual patient data from Kaplan–Meier plots was performed using two validated methods [24, 25] that were applied by our group in previous work [27, 28]: WebPlotDigitizer, Version 4.2 [22] and the R code of Guyot et al. [23].

From original trial reports, HRs with 95% confidence intervals (CI) as well as trial and patient characteristics were extracted. Accuracy of data extractions was checked by one investigator and double checked by another investigator. Divergences were resolved by group discussion. Risk of bias was appraised by one reviewer of our group according to recommendations from the Cochrane Collaboration’s revised tool for risk of bias assessment in randomised trials [38].

### Weibull model fit, estimates of absolute treatment effect, and numbers needed to treat notation

For estimation of survival functions and to achieve absolute risk differences of both treatment groups, parametric Weibull regression models were fitted for all trials separately [39]. For each individual trial, monthly probability differences (treatment–control) were estimated for being free of the analysed event from month 1 to the respective maximal observation time. To obtain estimates for monthly numbers needed to treat, these probability differences were inverted [40]. The number needed to treat is defined as the number of patients who need to be treated for a determined time interval to prevent one additional event in the treatment group in comparison to the placebo group. Hence, positive numbers needed to treat are indicative that the drug is beneficial. A neutral effect of a therapeutic intervention, corresponding to a HR of 1, is denoted by a value of infinity for the number needed to treat.

### Assessment of model validity

For appraisal of the validity of extracted data, comparison of HRs from the original papers to estimated Weibull HRs was carried out by calculating intra-class correlation coefficients. Furthermore, we plotted the estimated Weibull survival curves along with Kaplan–Meier survival curves from the extracted data for graphical assessment of the fit of the Weibull models.

### Meta-analysis

Random-effects inverse-variance meta-analysis for each single monthly time point was carried out separately to summarise numbers needed to treat overall as well as for the two drug classes; all trial data were included up to longest available follow-up. Primarily, all computations were performed on the probability difference scale. They were later transformed to the number needed to treat scale in order to display results in figures and graphs. For management and analysis of data, we used SAS (SAS Institute Inc., Cary, NC, USA), Version 9.4. All data will be made publicly available in an online repository after publication.

## Results

### Study selection

Original time-to-event information on a composite renal outcome was retrievable from Kaplan–Meier curves of 12 major cardiovascular outcome trials, which were included in the analysis: three trials investigated GLP-1 receptor agonists (AMPLITUDE-O [41], LEADER [42, 43], and REWIND [44, 45]), nine SGLT2 inhibitors (CANVAS [46], CREDENCE [47], DAPA-CKD [48], DECLARE-TIMI 58 [49], EMPA-KIDNEY [50], EMPA-REG [51, 52], EMPEROR-PRESERVED [15, 53], EMPEROR-REDUCED [12, 53], and VERTIS-CV [54]). For all digitized Kaplan–Meier plots, the number of patients at risk was reported. The EXSCEL trial reported results of exenatide on a composite renal outcome, however could not be included due to lack of Kaplan–Meier plots [55]. The SUSTAIN-6 trial was excluded because the extracted HRs were very far from the originally reported HR, suggesting methodological problems [56].

### Study characteristics

Clinical characteristics of the cardiovascular outcome trials’ study populations are listed in Table 2. In total, time-to-event information from 90,865 patients were extracted and used for further analysis. Median follow-up time ranged from 13.0 to 63.6 months among included studies; overall median follow-up time was 35.8 months. All cardiovascular outcome trials featured high cardiovascular risk populations [57]. The majority of trials were conducted in subjects selected for T2DM, while EMPEROR-PRESERVED [15] and EMPEROR-REDUCED [12] primarily included individuals with heart failure. DAPA-CKD [48] and EMPA-KIDNEY [50] were conducted in a population of subjects with CKD as the major inclusion criterion.

**Table 2:** Characteristics of included trials

Study	Journal and year	Study drug	Number of patients (n)	Mean age (years)	Male sex (%)	T2DM (%)	Mean HbA1c (%)	Mean BMI (kg/m2)	Mean diabetes duration (years)	Atherosclerotic cardiovascular disease (%)	Heart failure (%)	Hypertension (%)	Current smoker (%)	Mean eGFR (ml/min/1.73 m^2^)
GLP-1 receptor agonists														
AMPLITUDE -O [41]	NEJM 2021	Efpeglenatide	4076	64.5	77.0	100	8.9	32.7	15.4	n.a	18.1	91.3	15.4	72.4
LEADER [42, 43]	NEJM 2016	Liraglutide	9340	64.3	64.2	100	8.7	32.5	12.9	81.4	17.9	n.a	n.a	n.a
REWIND [44, 45]	Lancet 2019	Dulaglutide	9901	66.2	53.7	100	7.4	32.3	10.6	31.5	8.6	93.2	14.2	75.0
SGLT2 inhibitors														
CANVAS [46]	NEJM 2017	Canagliflozin	10 142	63.3	64.2	100	8.2	32.0	13.5	72.2	14.4	90.0	17.8	76.5
CREDENCE [47]	NEJM 2019	Canagliflozin	4401	63.0	66.1	100	8.3	31.4	15.7	50.4	14.8	96.8	14.5	56.2
DAPA-CKD [48]	NEJM 2020	Dapagliflozin	4304	61.9	66.9	67.5	n.a	29.5	n.a	n.a	10.9	n.a	13.6	43.1
DECLARE-TIMI58 [49]	NEJM 2019	Dapagliflozin	17 160	64.0	62.6	100	8.3	32.1	10.5	40.6	10.0	n.a	n.a	85.3
EMPA-KIDNEY [50]	NEJM 2023	Empagliflozin	6609	63.9	66.8	44.4	n.a	29.8	n.a	n.a	10.0	n.a	n.a	37.3
EMPA-REG [51, 52]	NEJM 2015	Empagliflozin	7020	63.1	71.5	100	8.1	30.7	n.a	75.6	10.1	95.1	n.a	74.0
EMPEROR-PRESERVED [15, 53]	NEJM 2021	Empagliflozin	5988	71.8	65.4	49.0	n.a	29.8	n.a	n.a	100	n.a	n.a	60.6
EMPEROR-REDUCED [12, 53]	NEJM 2020	Empagliflozin	3730	66.8	76.0	49.8	n.a	27.9	n.a	51.8	100	72.4	n.a	62.0
VERTIS-CV [54]	NEJM 2020	Ertugliflozin	8246	64.4	70.0	100	8.2	31.9	13.0	75.9	23.7	n.a	n.a	76.0

Characteristics of randomized controlled trials and their patient populations included in the meta-analysis

*T2DM* Type 2 diabetes mellitus, *GLP-1* glucagon-like peptide 1, *SGLT2* sodium-glucose transporter 2, *BMI* body mass index, *eGFR* estimated glomerular filtration rate, *n.a.* data not available

Mean estimated glomerular filtration rate (eGFR) at baseline ranged from 37.3 to 85.3 ml/min/1.73 m^2^. Definitions of composite renal endpoints were heterogeneous among the analysed cardiovascular outcome trials. However, all definitions included a pre-specified increase in serum creatinine or decrease in eGFR and incident end-stage kidney disease or renal replacement therapy. With the exception of AMLPITUDE-O [41], REWIND [44, 45], EMPEROR-PRESERVED [15], and EMPEROR-REDUCED [12], renal death was included in all composite renal endpoints. Risk of bias among all studies was low (Supplementary Table 1).

### Relative and absolute treatment effect estimates

**Fig. 1:**
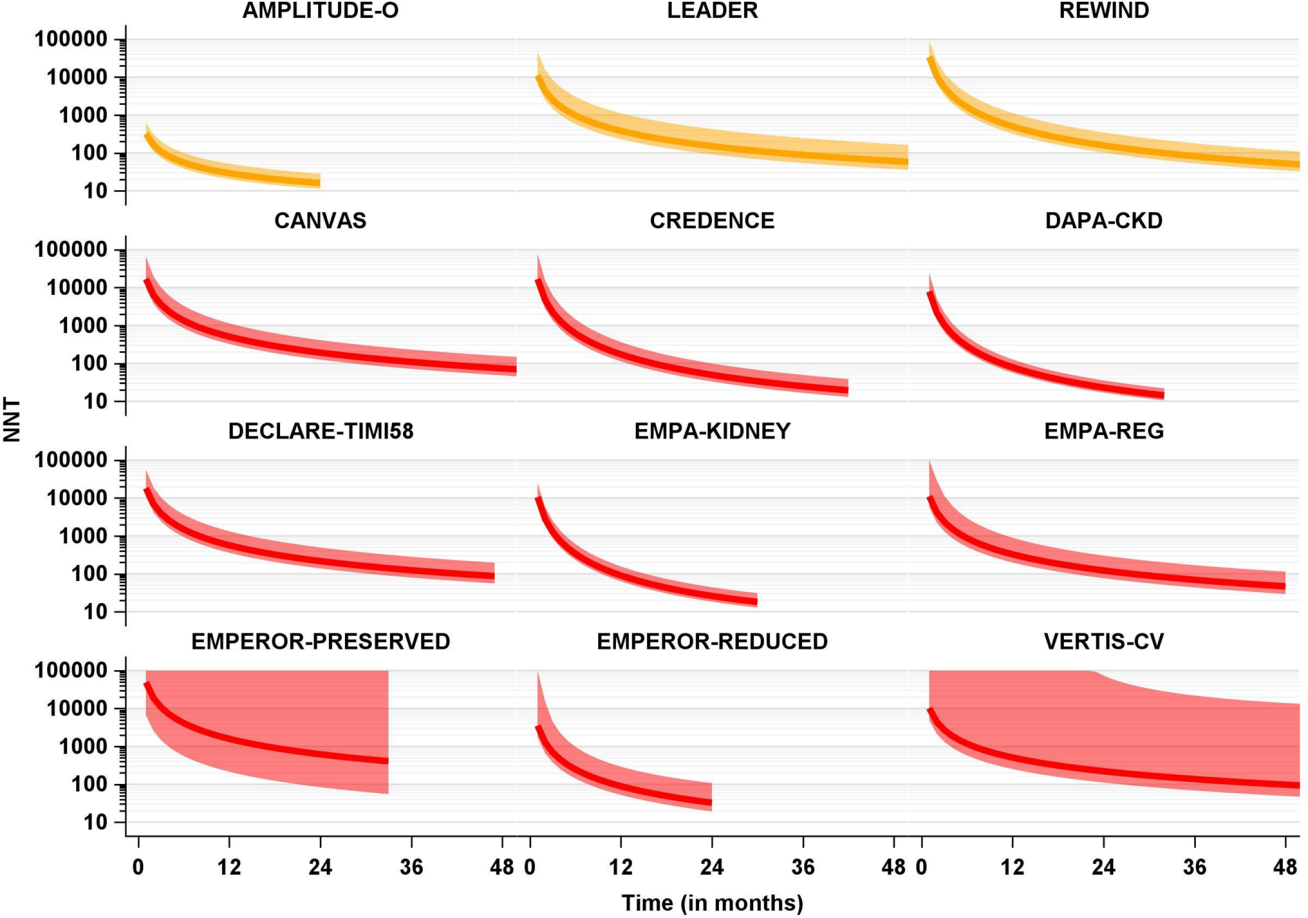
Numbers needed to treat in individual trials. Time-dependent numbers needed to treat (NNTs) over trial follow-up time for individual trials with their pointwise 95% confidence intervals (yellow: GLP-1 receptor agonists, red: SGLT2 inhibitors), Estimates and confidence intervals are truncated from above at 100,000

A total of 6199 (6.8%) patients experienced a composite renal event. Information on relative effect measures is given in Table 3: Original, digitalized, and Weibull HRs with 95% CI; absolute treatment effect estimates are reported as numbers needed to treat at 12, 24, 36, and 48 months. In addition, Fig. 1 depicts a graphical display of monthly number needed to treat point estimates with 95% CI for both trial drugs.

**Table 3:** Relative and absolute effect estimates for a composite renal outcome

Study	Number of events	Number of observations	Event proportion (%)	Median follow-up time (months)	Original HR [95% CI]	Extracted HR [95% CI]	Weibull HR [95% CI]	1 year NNT	2 years NNT	3 years NNT	4 years NNT
GLP-1 receptor agonists											
AMPLITUDE -O [41]	584	4076	14.3	21.0	0.68 [0.57; 0.79]	0.67 [0.57; 0.79]	0.67 [0.56; 0.78]	29 [20; 52]	16 [11; 28]	n.a	n.a
LEADER [42, 43]	603	9340	6.5	46.3	0.78 [0.67; 0.92]	0.78 [0.66; 0.92]	0.78 [0.65; 0.90]	382 [230; 1117]	151 [92; 427]	89 [54; 249]	61 [37; 172]
REWIND [44, 45]	1795	9901	18.1	63.6	0.85 [0.77; 0.93]	0.84 [0.77; 0.92]	0.84 [0.76; 0.92]	501 [322; 1128]	158 [102; 348]	82 [53; 180]	53 [34; 115]
SGLT2 inhibitors											
CANVAS [46]	260	10 142	2.6	29.0	0.60 [0.47; 0.77]	0.61 [0.47; 0.77]	0.62 [0.46; 0.77]	508 [327; 1141]	192 [126; 412]	110 [72; 232]	74 [48; 156]
CREDENCE [47]	374	4401	8.5	30.7	0.66 [0.53; 0.81]	0.66 [0.54; 0.82]	0.66 [0.53; 0.80]	173 [113; 365]	50 [33; 100]	25 [17; 50]	n.a
DAPA-CKD [48]	377	4304	8.8	25.5	0.56 [0.45; 0.68]	0.56 [0.45; 0.69]	0.56 [0.44; 0.67]	79 [57; 127]	23 [17; 36]	n.a	n.a
DECLARE-TIMI58 [49]	744	17 160	4.3	47.3	0.76 [0.67; 0.87]	0.76 [0.66; 0.88]	0.77 [0.66; 0.88]	566 [359; 1329]	217 [139; 496]	125 [80; 283]	n.a
EMPA-KIDNEY [50]	784	6609	11.9	22.9	0.71 [0.62; 0.81]	0.71 [0.62; 0.82]	0.71 [0.61; 0.81]	90 [63; 156]	26 [18; 44]	n.a	n.a
EMPA-REG [51, 52]	146	6968	2.1	34.9	0.54 [0.40; 0.75]	0.55 [0.40; 0.76]	0.55 [0.37; 0.73]	326 [199; 897]	123 [77; 304]	70 [44; 170]	47 [29; 115]
EMPEROR-PRESERVED [15, 53]	181	5988	3.0	19.5	0.95 [0.73; 1.24]	0.96 [0.71; 1.28]	0.96 [0.68; 1.23]	1585 [215; ∞; − 295]	619 [84; ∞; − 115]	n.a	n.a
EMPEROR-REDUCED [12, 53]	76	3730	2.0	13.0	0.51 [0.33; 0.79]	0.51 [0.32; 0.82]	0.51 [0.27; 0.75]	89 [52; 291]	33 [19; 108]	n.a	n.a
VERTIS-CV [54]	275	8246	3.3	36.1	0.81 [0.63; 1.04]	0.79 [0.62; 1.01]	0.78 [0.59; 0.96]	507 [253; 57892]	221 [111; 74212]	137 [69; 22076]	98 [49; 13985]

Effect estimates of individual trials for a composite renal outcome; depicted are raw event proportions, originally reported hazard ratios (*HR*) including 95% confidence intervals (*CI*), extracted HR from Kaplan–Meier plots, HR as calculated from the Weibull model, and number needed to treat (NNT) for 12–48 months

*GLP-1* glucagon-like peptide 1, *SGLT2* sodium glucose transporter 2

Accuracy of data extraction was assessed by a scatterplot (Supplementary Fig. 1) that compares the HR as reported from the original trial to the Weibull HR from the extracted data. Comparison revealed excellent correspondence indicated by an intra-class correlation of 99.5% (95% CI 99.0%; 100%). Supplementary Fig. 2 shows Kaplan–Meier survival curve estimates for both treatment groups in each trial along with the estimated survival curves from the corresponding Weibull models.

### Meta-analysis of absolute treatment effects

**Fig. 2:**
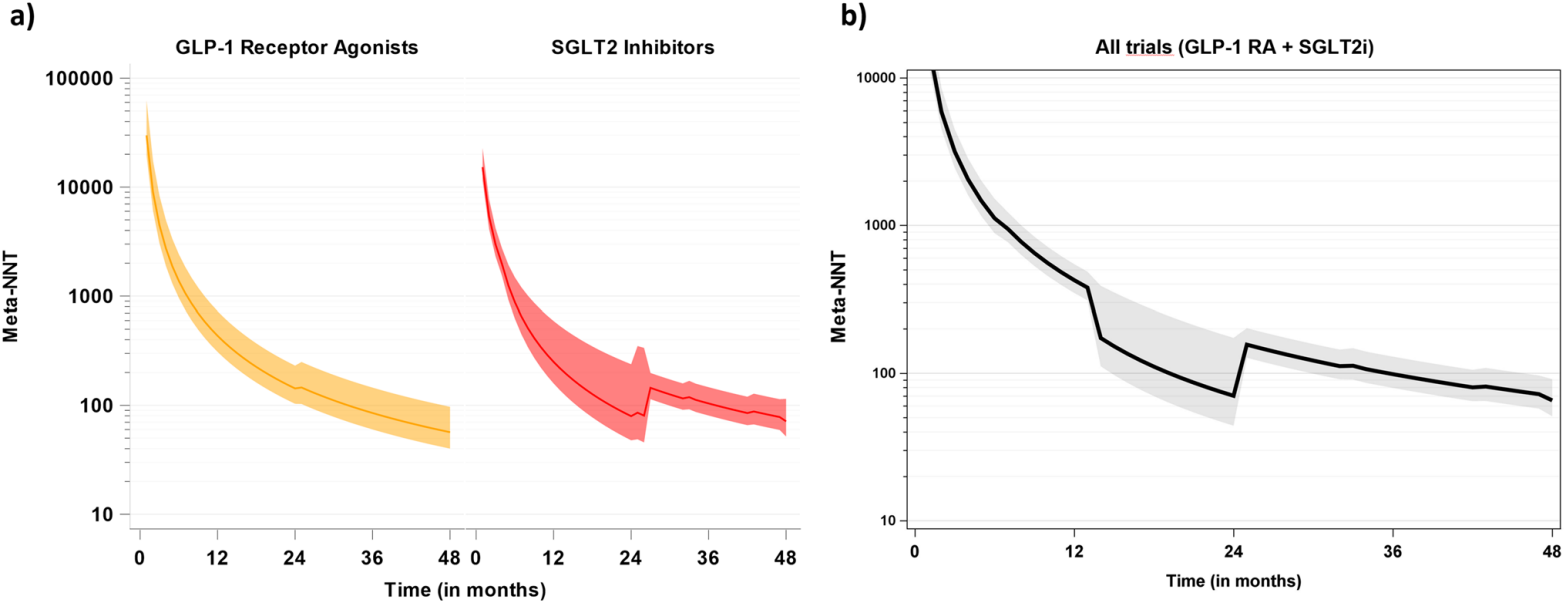
Meta-Analysis of numbers needed to treat. Random-effects inverse-variance meta-analysis of numbers needed to treat (Meta-NNTs, with 95% CI) over trial follow-up time. **a** Data were pooled from trials according to GLP-1 receptor agonists (yellow) or SGLT2 inhibitors (red) study drugs. **b** data were pooled from all trials, regardless of the tested study drug. Estimates and confidence intervals are truncated from above at 100,000 (**a**) and 10,000, respectively

Figure 2a shows treatment efficacy of GLP1 receptor agonists and SGLT2 inhibitors for the prevention of a single composite renal outcome on the number needed to treat scale: Estimated meta-numbers needed to treat were 85 (95% CI 60; 145) for GLP-1 receptor agonists and 104 (95% CI 81; 147) for SGLT2 inhibitors at the overall median follow-up time of 36 months. When pooling numbers needed to treat across both treatments, we found a meta-number needed to treat to prevent a single composite renal outcome of 65 (95% CI 51; 91) at a follow-up of 48 months (Fig. 2b).

## Discussion

In the present work, we performed a comprehensive meta-analysis of digitalized individual patient data from major cardiovascular outcome trials to assess absolute treatment effects (measured as numbers needed to treat) of GLP-1 receptor agonists and SGLT2 inhibitors on a composite renal outcome. For both drug classes, we observed a similar moderate absolute treatment efficacy when compared to placebo.

To improve cardiovascular outcomes, current guidelines encourage the use of GLP-1 receptor agonists and SGLT2 inhibitors in subjects with T2DM as well as SGLT2 inhibitors for the treatment of heart failure with the highest level of recommendation (Class I) [7, 58]. In subjects with T2DM and mild or moderate CKD, treatment with GLP-1 receptor agonists or SGLT2 inhibitors should be initiated, if therapy additional to metformin is required [58].

The majority of cardiovascular outcome trials on GLP-1 receptor agonists and SGLT2 inhibitors report treatment effects as relative measures (hazard, odds, or risk ratios), however, these ignore the baseline risk of the population when used as the main measure of efficacy. Higher baseline risk of a population is associated with higher absolute risk reductions, which can be achieved by an intervention, e.g. a drug treatment. Hence, guidelines for reporting randomised trials recommend providing information on relative and absolute effect measures, since absolute effect measures provide fundamental advantages for assessment of treatment efficacy and cost/benefit calculations [59]. They also facilitate comparative analyses with other drug classes and help patients to appraise expected benefits.

The quality of all cardiovascular outcome trials included in this analysis was high. These were well-conducted international randomised controlled trials published in high-impact journals with low risk of bias (Supplementary Table 1) [38]. Although inclusion criteria and target populations differed, cardiovascular risk in patients of all trials was high: populations featured either high proportions of cardiovascular disease, long-term T2DM or heart failure [12, 15, 41, 42, 44, 46–51, 54]. Average renal function was impaired in all populations, however, mean baseline eGFR notably differed from substantial CKD (< 60 mL/min/1.73 m^2^ in CREDENCE [47], DAPA-CKD [48], and EMPA-KIDNEY [50] to only mild renal impairment (60 to 89 mL/min/1.73 m^2^) in the other cardiovascular outcome trials [12, 15, 41, 42, 44, 46, 49, 51, 54]. Subjects with a lower eGFR are at higher risk for progression of kidney disease, indicating a baseline heterogeneity of renal risk in the analysed populations [60]. This emphasises the importance of looking at relative as well as absolute treatment effects. Relative treatment effects (HRs) for a composite renal outcome were comparable in CANVAS (0.60) [46], CREDENCE (0.66) [47], and DAPA-CKD (0.56) [48], but differed markedly on the number needed to treat scale: The 3-year meta-number needed to treat in CANVAS was 110, but 25 in CREDENCE; the 2-year meta-number needed to treat was 192 in CANVAS, but 23 in DAPA-CKD—indicating a substantially higher absolute efficacy in higher-risk patient populations.

Compared to the efficacy of SGLT2 inhibitors on hospitalisation for heart failure in DAPA-HF [11, 27] (2-year meta-number needed to treat of 21) and EMPEROR-REDUCED [12, 27] (2-year meta-number needed to treat of 15), the present analysis revealed only moderate absolute beneficial treatment effects of GLP-1 receptor agonists (36-month meta-number needed to treat of 85) and SGLT2 inhibitors (36-month meta-number needed to treat of 104) for a composite renal outcome. Whereas SGLT2 inhibitors are preferable over GLP-1 receptor agonists in heart failure with or without T2DM due to results from specific heart failure trials and current guideline recommendations, our analysis could not identify a greater advantage for either drug class regarding composite renal outcomes. However, appraisal of clinical relevance of a drug class in different populations based on comparing treatment efficacy for different outcomes is difficult and should not guide clinical decisions.

Type 2 diabetes mellitus is the leading cause of CKD [60]. It is associated with high morbidity/mortality burden and health expenditures [61]. There has been discussion about optimal selection of clinically relevant endpoints in trials to assess kidney-specific drug efficacy [62, 63]. Incident end-stage kidney disease or need for renal replacement therapy is without doubt a very serious clinical condition. Treatment of patients with end-stage kidney disease or renal transplantation is complex, logistically challenging, and causes substantial health expenditures [64–67]. Among cardiovascular outcome trials in T2DM, incident end-stage kidney disease is rare with event proportions of less than one percent, which generates the need to evaluate a composite outcome for renal drug efficacy in trials with reasonable sample size and duration of follow-up [68]. A decline of kidney function to a non-end-stage extent (measured as increase in serum creatinine or decline in eGFR) is the additional outcome that was included in all of the composite endpoints of cardiovascular outcome trials in the present analysis. However, definitions ranged from sustained ≥ 30% decline in eGFR (REWIND [44]) to doubling of serum creatinine [43, 46, 47, 52, 54], which approximates a 57% decline in eGFR and is a long-established kidney outcome, which is highly predictive of end-stage kidney disease (Table 1) [69]. The threshold of ≥ 40% eGFR decline, as applied in five cardiovascular outcome trials of the present analysis [12, 15, 41, 49, 50], was a reliable predictor of end-stage kidney disease or the traditional doubling of serum creatinine (HR ~ 20 over a median follow-up of two years) [70], whereas the association of a threshold of ≥ 30% serum creatinine increase was less strong (HR ~ 9). Hence, despite heterogeneity among composite renal outcome definitions, we assume all singular composite endpoint definitions include clinically meaningful outcomes suitable to assess kidney-specific treatment benefits in clinical trials (Table 1).

Renal pathogenesis includes inflammatory, humoral, metabolic and oxidative stress factors as well as macro- and microvascular disease [71]. Together with blood pressure reduction [72], intensive glycaemic control showed beneficial effects on diabetic kidney disease [73]. Metformin, the long-term first choice for treating people with T2DM has a 10-year number needed to treat of ~ 10–20 for major cardiovascular or diabetes-related endpoints [74, 75]. Due to consistent treatment effects of GLP-1 receptor agonists and SGLT2 inhibitors on major adverse cardiovascular events, and of SGLT2 inhibitors on renal endpoints with and without concomitant metformin use [76, 77], recent concepts have promoted the use of GLP1-receptor agonists and SGLT2 inhibitors in subjects with T2DM and CKD regardless of metformin administration [78]. However, the nephroprotective potential of GLP-1 receptor agonists and SGLT2 inhibitors is not limited to improved glycaemic control. Several indirect (e.g. blood pressure in SGLT2 inhibitors, weight loss in GLP-1 receptor agonists) and direct effects on the kidney have been reported (e.g. improvement of intrarenal haemodynamics or prevention of ischaemic and oxidative damage in SGLT2 inhibitors) [79, 80]. Positive results of DAPA-CKD [48] and EMPA-Kidney [50], the two large scale randomised controlled trials of SGLT2 inhibitors in subjects with pre-existing impaired renal function as the primary inclusion criterion, encourage the use of SGLT2 inhibitors in individuals with kidney disease regardless of coexisting T2DM. Similar trials of GLP-1 receptor agonists are currently not available. However, according to our results, they may represent an important area of future research.

In addition to previous analyses of other outcomes, this study now shows meta-analysed absolute effects of these drug classes regarding number needed to treat for a composite renal outcome. Ludwig et al. reported numbers needed to treat for major adverse cardiovascular events [40] and Davies et al. analysed a primary composite outcome and all‐cause mortality [81]. Our group previously analysed the trials´ primary outcomes, all-cause and cardiovascular mortality, as well as hospitalisation for heart failure [27, 28]. Although other groups used different digitalization tools and statistical methods, results for the analysed outcomes were comparable, which demonstrates that the method of digitalization of individual patient data to assess absolute treatment effects is valid and reliable, especially for cardiovascular outcome trials.

Due to our prespecified selection criteria, no systematic literature review was conducted. Trial selection for our meta-analysis relied on a shared definition of cardiovascular outcome trials as reported in the annual cardiovascular outcome trial summit reports [29–35] and thus the most important studies in the field were included; however, we cannot rule out that smaller non-cardiovascular outcome trial studies with renal outcomes may have been overlooked. The exclusion of trials (e.g. EXSCEL) due to lack of Kaplan–Meier plots may also have introduced bias. Differences in characteristics and design of included cardiovascular outcome trials introduce heterogeneity, a general limitation of meta-analyses requiring a cautious interpretation of results. Within the present work, heterogeneity derives from differences in baseline kidney function, heterogeneous baseline risk for the analysed outcome as well as differences in definitions of the outcomes among individual trials, all of which may introduce bias. However, we believe all composite renal outcome definitions among the included cardiovascular outcome trials to be clinically meaningful and suitable to assess drug efficacy.

Additionally, computations within the present work rely on digitalized individual patient outcomes and fitted Weibull models not including original patient data, which introduces risk of differences between original and extracted event counts (Supplementary Table 2) and does not allow appropriate interaction analyses. Therefore, no further insight into associations of patient characteristics with outcomes other than the analysed composite outcome can be obtained. The inability to perform subgroup analyses prevents the identification of effect modifiers. However, lack of access to individualised patient data renders the method of data digitalization unavoidable to perform the present analysis of absolute treatment effects. Sensitivity analyses, in the form of assessment of the Weibull curves (direct origin of estimated number needed to treat) fit, show excellent alignment to extracted data (Supplementary Fig. 2), which strongly indicates the validity of the applied methods.

## Conclusions

The present meta-analysis of digitalized individual patient data revealed moderate and similar absolute treatment benefits of GLP-1 receptor agonists and SGLT2 inhibitors compared to placebo for a composite renal outcome.

### Supplementary Information

Below is the link to the electronic supplementary material.

Supplementary file1 (PDF 342 kb)

## Data Availability

All data will be made publicly available in an online repository after publication.
