# Supplementary material for: Absolute treatment effects of novel antidiabetic drugs on a composite renal outcome: meta-analysis of digitalized individual patient data
Source: J Nephrol. 2024 Jan 18;37(2):309–21. doi: 10.1007/s40620-023-01858-8 (PMC11043149; doi:10.1007/s40620-023-01858-8)
Supplement: Supplementary file 1 — Supplementary file1 (PDF 342 kb) [file 40620_2023_1858_MOESM1_ESM.pdf]

Supplementary Figure 1 – Scatterplot evaluation of the Weibull model fit

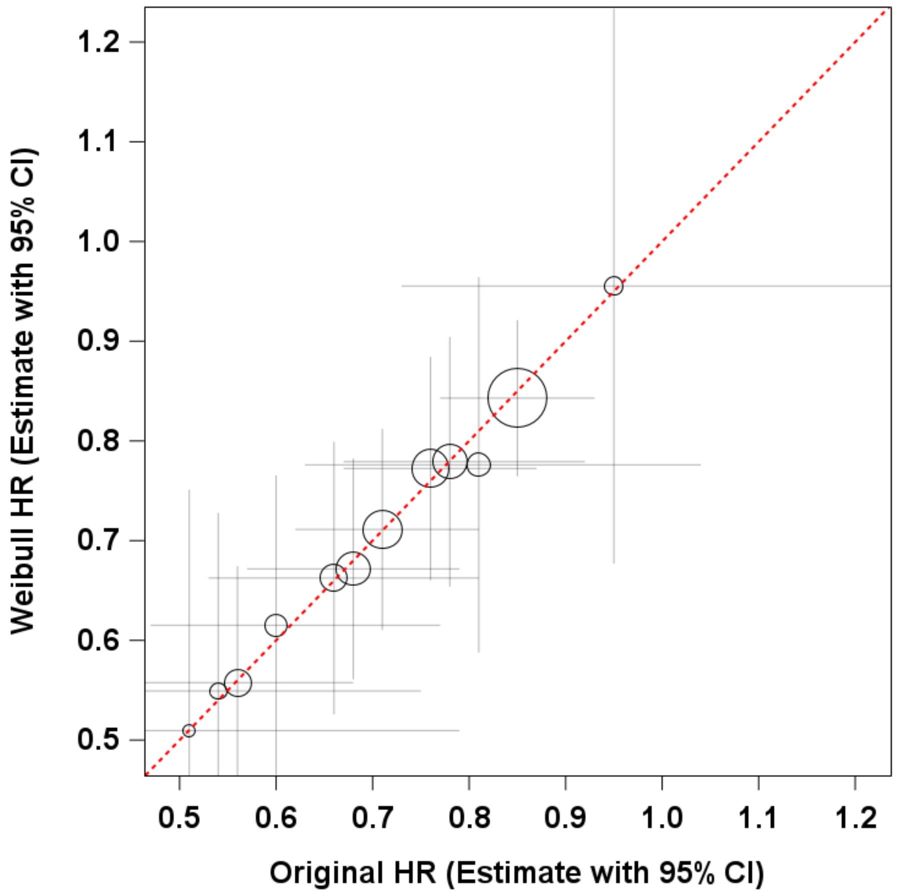

Supplementary Figure 1: Scatterplot to compare the hazard ratios (HR) from the original publications (x-axis, with 95% CI) with the hazard ratios from the fitted Weibull model of the extracted data (y-axis, with 95% CI)

Supplementary Figure 2 – Kaplan-Meier evaluation of the Weibull model fit

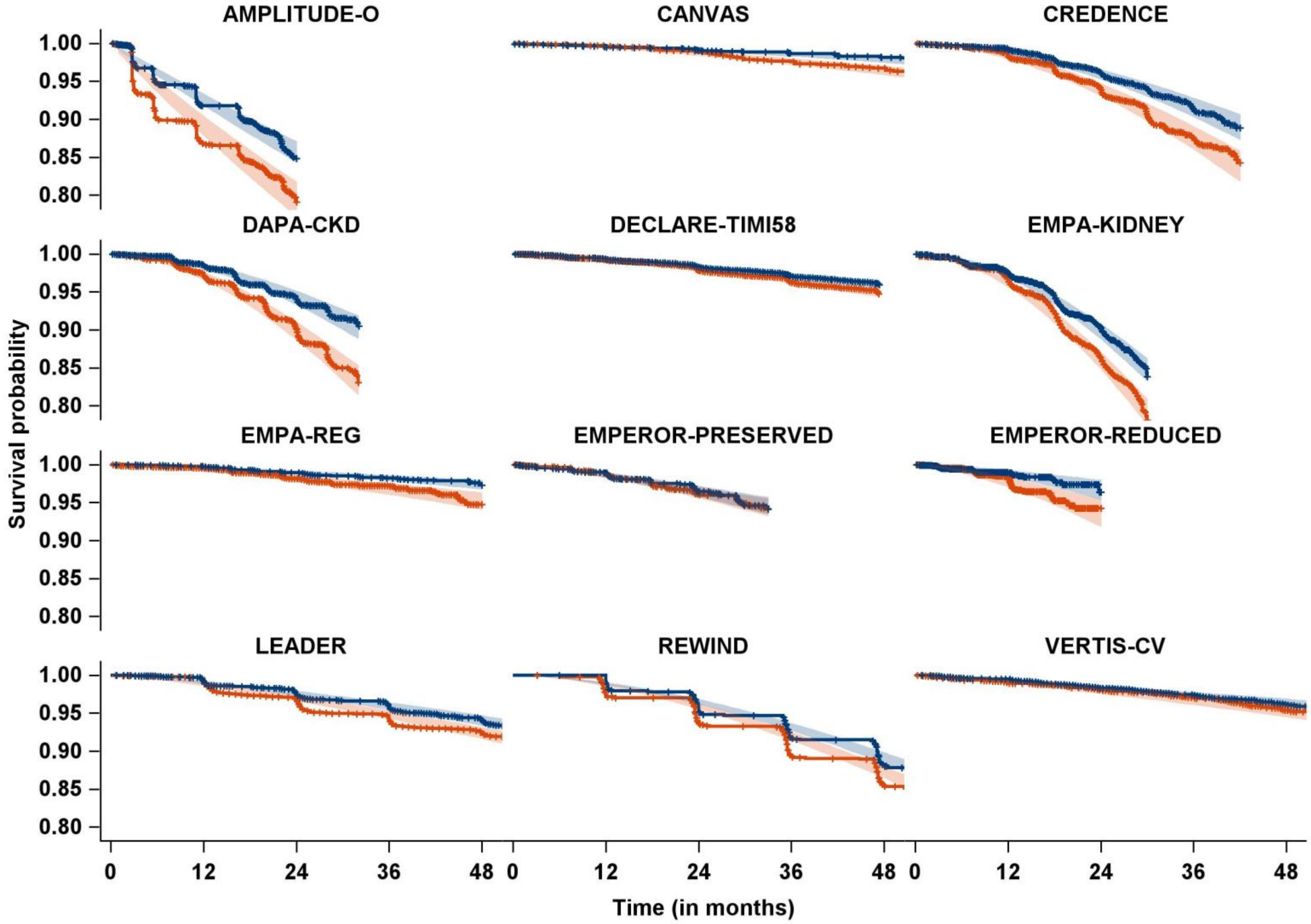

Supplementary Figure 2: Kaplan-Meier survival curve estimates for both treatment groups along with the estimated survival curves from the corresponding Weibull models with 95% confidence intervals (blue: treatment group, red: placebo group)

Supplementary Table 1 – Risk of bias in included trials

Study	Randomization process	Deviations from intended interventions	Missing outcome data	Measurement of the outcome	Selection of the reported result	Overall risk of bias
AMPLITUDE -O [41]	+	+	+	+	+	+
LEADER [42,43]	+	+	+	+	+	+
REWIND [44,45]	+	+	+	+	+	+
CANVAS [46]	+	+	+	+	+	+
CREDENCE [47]	+	+	+	+	+	+
DAPA-CKD [48]	+	+	+	+	+	+
DECLARE-TIMI58 [49]	+	+	+	+	+	+
EMPA-KIDNEY [37]	+	+	+	+	+	+
EMPA-REG [50,51]	+	+	+	+	+	+
EMPEROR-PRESERVED [15,52]	+	+	+	+	+	+
EMPEROR-REDUCED [12,52]	+	+	+	+	+	+
VERTIS-CV [53]	+	+	+	+	+	+

+ low risk of bias ? unclear risk of bias − high risk of bias

Supplementary Table 1: Risk of bias assessment of all included trials, according to the Cochrane Collaboration's revised tool for assessing risk of bias in randomised trials [38].

Supplementary Table 2 – Extracted and original number of events, relative and absolute effect estimates for a composite renal outcome

Study	Number of Events	Number of Observations	Event Proportion (%)	Number of Events Verum Extracted	Number of Events Placebo Extracted	Number of Events Verum Original	Number of Events Placebo Original	Original HR [95% CI]	Extracted HR [95% CI]	Weibull HR [95% CI]
AMPLITUDE -O [41]	584	4076	14.3	343	241	353	250	0.68 [0.57; 0.79]	0.67 [0.57; 0.79]	0.67 [0.56; 0.78]
LEADER [42, 43]	603	9340	6.5	267	336	268	337	0.78 [0.67; 0.92]	0.78 [0.66; 0.92]	0.78 [0.65; 0.90]
REWIND [44, 45]	1795	9901	18.1	834	961	848	970	0.85 [0.77; 0.93]	0.84 [0.77; 0.92]	0.84 [0.76; 0.92]
CANVAS [46]	260	10 142	2.6	134	126	Absolute numbers not given		0.60 [0.47; 0.77]	0.61 [0.47; 0.77]	0.62 [0.46; 0.77]
CREDENCE [47]	374	4401	8.5	152	222	153	224	0.66 [0.53; 0.81]	0.66 [0.54; 0.82]	0.66 [0.53; 0.80]
DAPA-CKD [48]	377	4304	8.8	138	239	142	243	0.56 [0.45; 0.68]	0.56 [0.45; 0.69]	0.56 [0.44; 0.67]
DECLARE-TIMI58 [49]	744	17 160	4.3	326	418	370	480	0.76 [0.67; 0.87]	0.76 [0.66; 0.88]	0.77 [0.66; 0.88]
EMPA-KIDNEY [37]	784	6609	11.9	329	455	384	504	0.71 [0.62; 0.81]	0.71 [0.62; 0.82]	0.71 [0.61; 0.81]
EMPA-REG [50, 51]	146	6968	2.1	78	68	70	60	0.54 [0.40; 0.75]	0.55 [0.40; 0.76]	0.55 [0.37; 0.73]
EMPEROR-PRESERVED [15, 52]	181	5988	3.0	89	92	108	112	0.95 [0.73; 1.24]	0.96 [0.71; 1.28]	0.96 [0.68; 1.23]
EMPEROR-REDUCED [12, 52]	76	3730	2.0	26	50	30	58	0.51 [0.33; 0.79]	0.51 [0.32; 0.82]	0.51 [0.27; 0.75]
VERTIS-CV [53]	275	8246	3.3	168	107	175	108	0.81 [0.63; 1.04]	0.79 [0.62; 1.01]	0.78 [0.59; 0.96]

Supplementary Table 2: Extracted and original numbers of events in overall study populations, and in verum and placebo groups. Effect estimates of individual trials for a composite renal outcome; depicted are raw event proportions, originally reported hazard ratios (HR) including 95% confidence intervals (CI), extracted HR from Kaplan-Meier plots, HR as calculated from the Weibull model. GLP-1 = glucagon-like peptide 1; SGLT2 = sodium glucose transporter 2.
